# Supplementary material for: Enhanced Cerebral Blood Volume under Normobaric Hyperoxia in the J20-hAPP Mouse Model of Alzheimer’s Disease
Source: Sci Rep. 2020 May 5;10:7518. doi: 10.1038/s41598-020-64334-4 (PMC7200762; doi:10.1038/s41598-020-64334-4)
Supplement: Supplementary file 1 — Supplementary information. [file 41598_2020_64334_MOESM1_ESM.pdf]

Supplemental Data

Enhanced Cerebral Blood Volume under Normobaric Hyperoxia in the J20-hAPP Mouse Model of Alzheimer's Disease

Osman Shabir, Paul Sharp, Monica A Rebollar, Luke Boorman, Clare Howarth, Stephen B Wharton, Sheila E Francis & Jason Berwick

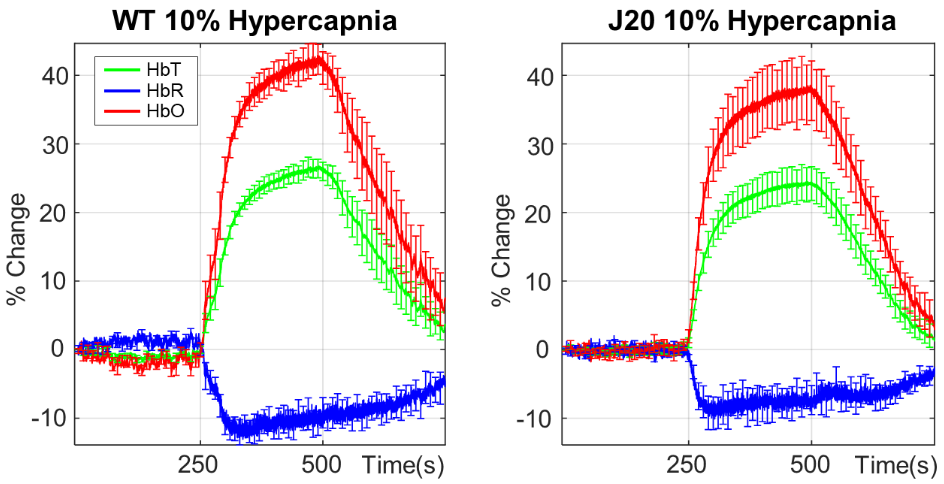

Figure S1 – 10% Hypercapnia Responses. Chronic data for WT (n=8) and J20-hAPP (n=9) hypercapnia responses showed no significant differences between groups. Hypercapnia was performed in 100% oxygen.

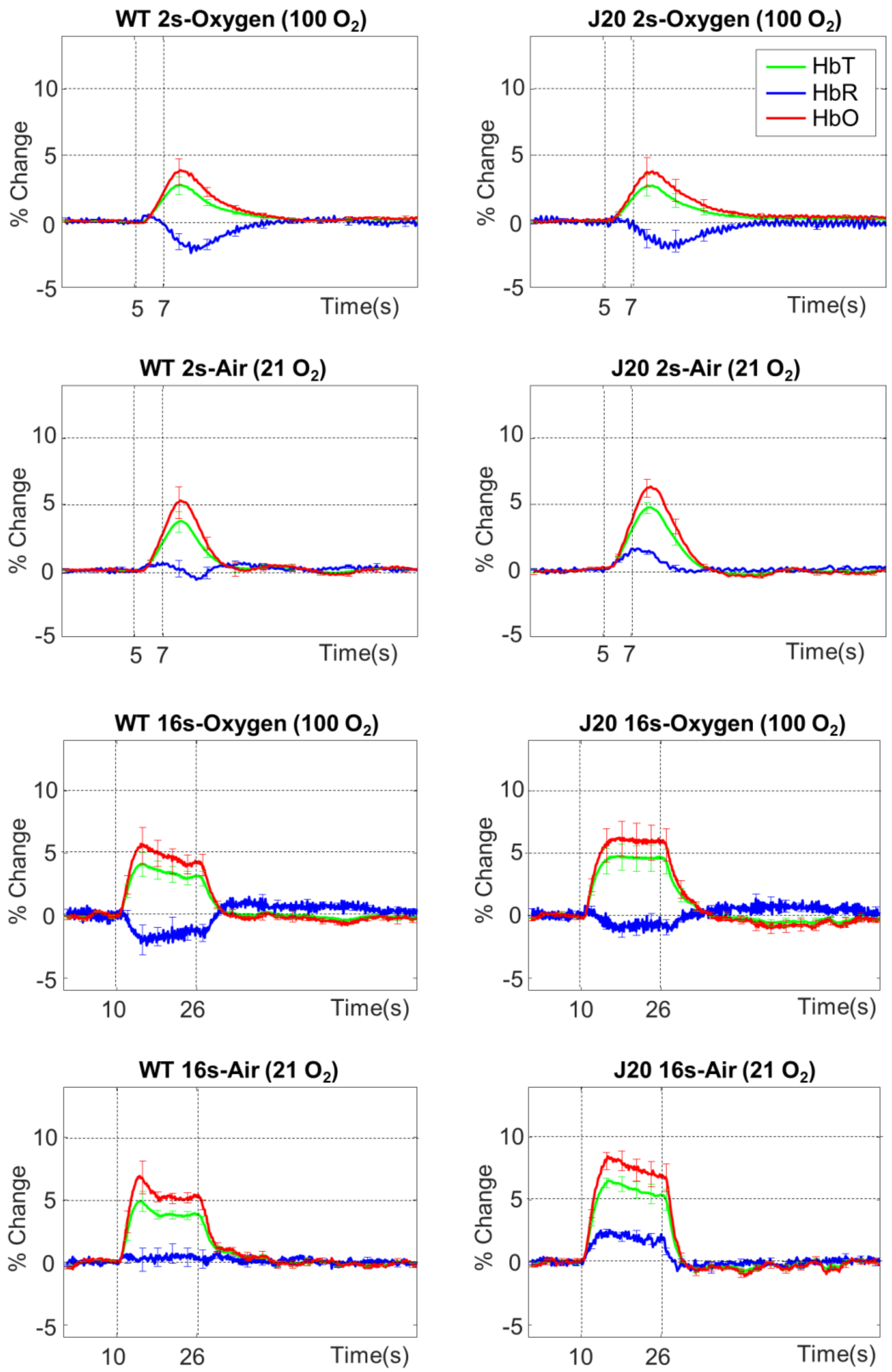

Figure S2 – Haemodynamic Responses During Acute Imaging Session (with Electrode Inserted). There are no significant differences in HbT between WT (n=6) and J20-hAPP (n=5). HbT/blood volume recovers in both groups as a result of time post-electrode insertion. Washout of HbR is impaired under 21% oxygen conditions in both WT and J20-hAPP mice.
